# Effect of Imaging Parameter Thresholds on MRI Prediction of Neoadjuvant Chemotherapy Response in Breast Cancer Subtypes

**DOI:** 10.1371/journal.pone.0142047

**Published:** 2016-02-17

**Authors:** Wei-Ching Lo, Wen Li, Ella F. Jones, David C. Newitt, John Kornak, Lisa J. Wilmes, Laura J. Esserman, Nola M. Hylton

**Affiliations:** 1 Department of Radiology and Biomedical Imaging, University of California San Francisco, San Francisco, California, United States of America; 2 Department of Epidemiology and Biostatistics, University of California San Francisco, San Francisco, California, United States of America; 3 Department of Surgery and Radiology, University of California San Francisco, San Francisco, California, United States of America; University of Algarve, PORTUGAL

## Abstract

The purpose of this study is to evaluate the predictive performance of magnetic resonance imaging (MRI) markers in breast cancer patients by subtype. Sixty-four patients with locally advanced breast cancer undergoing neoadjuvant chemotherapy were enrolled in this study. Each patient received a dynamic contrast-enhanced (DCE-MRI) at baseline, after 1 cycle of chemotherapy and before surgery. Functional tumor volume (FTV), the imaging marker measured by DCE-MRI, was computed at various thresholds of percent enhancement (PE_t_) and signal-enhancement ratio (SER_t_). Final FTV before surgery and percent changes of FTVs at the early and final treatment time points were used to predict patients’ recurrence-free survival. The full cohort and each subtype defined by the status of hormone receptor and human epidermal growth factor receptor 2 (HR+/HER2-, HER2+, triple negative) were analyzed. Predictions were evaluated using the Cox proportional hazard model when PE_t_ changed from 30% to 200% in steps of 10% and SER_t_ changed from 0 to 2 in steps of 0.2. Predictions with high hazard ratios and low p-values were considered as strong. Different profiles of FTV as predictors for recurrence-free survival were observed in each breast cancer subtype and strong associations with survival were observed at different PE_t_/SER_t_ combinations that resulted in different FTVs. Findings from this retrospective study suggest that the predictive performance of imaging markers based on FTV may be improved with enhancement thresholds being optimized separately for clinically-relevant subtypes defined by HR and HER2 receptor expression.

## Introduction

Breast cancer is a heterogeneous disease comprising subtypes with different risks for progression and recurrence and with different treatment outcomes [1]. Breast cancer subtypes defined by hormone receptor (HR) and human epidermal growth factor receptor 2 (HER2) status have been shown as not only having distinctive molecular phenotypes [2], but also having different prognoses [3]. With the understanding of subtype classifications, targeted therapies have been successfully employed in breast cancer, leading to significant improvements in disease-free survival [4]. Hormonal treatment is now commonly recommended to patients with HR+ tumors, whereas several anti-HER2 therapies are available for patients with HER2+ tumors. Given the lack of hormone receptors and HER2, patients with basal like tumors, also known as triple negative (TN) breast cancer, have limited therapeutic options with relatively poor outcomes. Thus, the development of improved treatment for TN patients is currently an important area of research.

In recent years, there has been a shift to greater use of neoadjuvant chemotherapy (NACT) prior to surgery. While the administration of NACT showed no difference in disease-free or overall survival compared to traditional adjuvant chemotherapy [5,6], NACT not only enables tumor downgrade for breast conservation surgery, it also provides means to evaluate the effectiveness of chemotherapy in patients.

Dynamic contrast-enhanced magnetic resonance imaging (DCE-MRI) is a noninvasive imaging technique that can be used as an imaging marker for characterizing tumor response to therapy [7–10]. Several clinical studies in the NACT setting have demonstrated that tumor reduction measured by DCE-MRI is in concordance with pathologic response, and the measurement can be a prognostic indicator for survival [11–14]. Concordance has been shown to vary by tumor subtype and MRI phenotype, with higher concordance in well-defined HER2+ and triple negative breast cancer but lower in diffuse HR+ tumors [15].

Functional tumor volume (FTV) calculated from DCE-MRI is one of the predictors for treatment response. It is computed based on the minimum threshold for the early percent enhancement (PE) after contrast injection and the early-to-late signal enhancement ratio (SER) in DCE-MRI [13]. Change in FTV can be used to monitor tumor regression in breast cancer patients undergoing NACT. Our previously published work showed that, FTV changes measured between pre-treatment MRI (baseline) and MRIs at the early treatment and before surgery, was a predictor for recurrence-free survival (RFS) [13]. In a separate study, we found that imaging metrics (i.e. minimum thresholds for percent enhancement (PE_t_) and signal enhancement ratio (SER_t_)) had a substantial influence on the FTV calculation and a strong effect on the resulting FTV-RFS association [16].

Given the different MRI enhancement patterns among subtypes and breast MR phenotypes, we hypothesized that optimization of PE and SER thresholds by subtype could further improve the predictive ability of FTV. In the present study, we extended the previous work by exploring the prediction profile of FTV in clinically-relevant subtypes defined by the HR and HER2 status and using various forms of FTV measured at early and late treatment time points as predictors. By using the same patient cohort, not only that we were able to compare findings from the present study to the previous work, we were also able to assess the influence of PE_t_ and SER_t_ on FTV survival prediction in each breast cancer subtype compared to the full cohort.

## Materials and Methods

### Ethics Statement

Sixty-eight patients with stage II or III locally advanced breast cancer were enrolled in a neoadjuvant chemotherapy breast cancer protocol. The protocol along with the consent procedure was reviewed by the University of California San Francisco institutional review board (IRB) and approved by the Committee of Human Research (CHR) under the University of California San Francisco Human Research Protection Program between 1995 and 2002. All patients had given their written informed consent to participate this study. The image analysis for this retrospective study was also approved by the University of California San Francisco IRB-CHR.

### Patient Population

All patients had confirmed breast cancer diagnoses based on histopathology of biopsy or surgical excision, and none had prior treatment with chemotherapy, surgery or radiation. All patients received pre-operative chemotherapy with four cycles of adriamycin-cytoxan administered every three weeks. A subset of patients received additional weekly treatment with taxane. DCE-MRI scans were scheduled for baseline (MRI_1_), after one cycle of chemotherapy (MRI_2_), inter-regimen (MRI_3_, taxane receivers only) and at the completion of chemotherapy prior to surgery (MRI_4_). Because treatment length and regimen varied among patients, the MRI exam performed after all NACT and before surgery was designated as final MRI (MRI_f_).

Recurrence-free survival (RFS) was assessed for each patient at 6-month or 1-year intervals following surgery upon March 2008. For patients that recurred, length of RFS was defined as the time from surgery to either local or distant recurrence. Patients that were lost to follow-up or did not recur at their most recent follow-up were considered censored and length of RFS was defined by the time to the most recent follow-up. Patient age, lesion characteristics including pretreatment tumor size, histologic type, pathologic grade, tumor subtype, recurrence type (local or distant) and time to RFS (or time to follow up for patients with no recurrence) were recorded.

### MRI Acquisition

Breast MRI was acquired on a 1.5-T scanner (Signa, GE Healthcare, Milwaukee, WI) using a bilateral phased array breast coil. The MR imaging protocol included a 3D localizer sequence and a contrast-enhanced sequence using a high spatial resolution, low temporal resolution T1-weighted pulse sequence developed for pre-surgical staging. For the contrast-enhanced sequence, unilateral sagittal images of the tumor bearing breast (ipsilateral) were obtained using a fat-suppressed T1-weighted 3D fast gradient-recalled echo sequence with high spatial resolution (TR/TE, 8/4.2; flip angle, 20 degrees; field of view, 18–20 cm; acquisition matrix, 256 x 192 x 60, section thickness, 2 mm; spatial resolution, 0.7 x 0.94 x 2.0 mm^3^) [17]. The contrast agent, gadopentetate dimeglumine (Magnevist, Bayer HealthCare, Berlin, Germany), was injected at a dose of 0.1 mmol/kg of body weight (1.2 mL per second) followed by a 10 mL saline flush. Three time points were acquired during each contrast-enhanced MRI protocol: a pre-contrast scan (t_0_), followed by 2 time points measured in the early (t_1_) and late phases (t_2_) after contrast injection. Imaging time was approximately 5 minutes per acquisition, resulting in effective early and late post-contrast time points of 2.5 minutes and 7.5 minutes from the start of the scan, respectively, using standard k-space sampling [13]. Fat suppression was used to eliminate the bright fat signal surrounding enhancing lesions on the T1-weighted MRI and was performed using a frequency-selective inversion recovery preparatory pulse.

### Image Analysis

MR images were analyzed to measure tumor volume using a semi-automated software algorithm developed in the IDL programming environment (ITT Visual Information Solutions, Boulder, CO) at our institution [18]. Each 3D volume of interest (VOI) enclosing all strongly enhancing regions was manually defined by a trained researcher by placing rectangular regions of interest (ROI) on two orthogonal maximum intensity projection (MIP) images created from the early post-contrast scan. Intersection of the two projected ROIs was used to define the VOI. Obvious enhanced non-cancerous regions intruding on the VOI, such as vessels or the heart, were eliminated manually by drawing an irregular ROI. All subsequent steps in the analysis were fully automated.

### Percent Enhancement (PE) and Signal Enhancement Ratio (SER) Analysis

PE and SER are used in DCE-MRI to measure uptake and washout rate of the contrast agent by modeling the time-signal intensity curve after the injection [16]. Early (PE_early_) and late (PE_late_) percent enhancement values were calculated as ${\text{PE}}_{\text{early}}=\frac{S_{E}-S_{0}}{S_{0}}x100\%$; ${\text{PE}}_{\text{late}}=\frac{S_{L}-S_{0}}{S_{0}}x100\%$ where S_0_, S_E_ and S_L_ represent the pre-contrast, early post-contrast, and late post-contrast signal intensity values, respectively. SER was defined as the ratio of PE_early_ to PE_late_ ($\text{SER}=\frac{S_{E}-S_{0}}{S_{L}-S_{0}}$) [17]. High SER value is indicative of tissue with a strong signal washout characteristic [19].

FTV was calculated as the volume of all voxels within the VOI exceeding thresholds for PE and SER. A minimum early enhancement threshold, PE_t_, was applied to the PE map followed by a connectivity test to eliminate very small regions, creating a final enhancing tissue mask. SER was then calculated for all voxels in the mask and a minimum signal enhancement ratio threshold, SER_t_, was applied to SER values within the VOI to calculate FTV.

For this study, we automated the optimization process using an in-house software developed using MATLAB (version R2012b, The MathWorks, Natick, MA). We investigated ranges of PE_t_ from 30% to 200% in steps of 10% and SER_t_ from 0.0 to 2.0 in steps of 0.2. Changes from baseline at the early treatment (ΔFTV_2_) and after NACT (ΔFTV_f_), and absolute FTV after NACT (FTV_f_), were computed and tested as predictors. The default setting was PE_t_ = 70% and SER_t_ = 0, which had been used on typical FTV calculations [13,16]. In this paper, the effect of changing PE_t_ and SER_t_ on FTV predictions was compared to that calculated from the default setting.

### Tumor Subtype Assessment

Tumor hormone receptor (HR) and HER2 status were extracted from pathology reports. Estrogen or progesterone receptor status was positive at immunohistochemical staining of 1% or more tumor cells, and HER2 status was positive on an immunohistochemical score of 3+ or a fluorescence in situ hybridization of HER2-to-chromosome 17 centromere ratio greater than 2.2. We defined triple-negative (TN) disease as breast cancer negative for estrogen receptor, progesterone receptor and HER2 by following that assessment. Based on the tumor HR and HER2 status defined above, the full cohort of our patient population (n = 64) was subset into HR+/HER2-, HER2+, TN, and unknown groups, in which HER2+ included both HR+/HER2+ and HR-/HER2+ patients.

### Statistical Analysis and Data Visualization

Cox proportional hazard models [20] were fitted to the FTV predictors and RFS for each combination of PE_t_ and SER_t_ to estimate the association between FTV and RFS. Estimated hazard ratios per unit change in the predictors ΔFTV_2_, ΔFTV_f_, and FTV_f_ were generated in each model for the full patient cohort and within subsets defined by tumor subtypes (HR+/HER2-, HER2+, TN), along with Wald 95% confidence intervals (CI) and likelihood ratio test p-values. The cutoff of *p* < 0.05 was used to differentiate PE_t_/SER_t_ values with higher predictive performance. All statistical computations were conducted using the R statistical analysis software package [21] and the survival library therein.

Estimated hazard ratios / p-values of all PE_t_/SER_t_ combinations tested for each predictor in subtype or full cohort were visualized as heat maps. Each heat map was generated based on a grid composed of PE_t_ in the y-axis ranging from 30% to 200% in steps of 10% and SER_t_ in the x-axis ranging from 0 to 2.0 in steps of 0.2. Each PE_t_/SER_t_ combination was used to calculate the corresponding FTV predictors. The subsequent heat maps of estimated hazard ratios and p-values for prediction of RFS were generated using the filled contour function in MATLAB (MATLAB Release 2012b, The MathWorks, Inc., Natick, Massachusetts, United States). The optimized PE_t_/SER_t_ combination was chosen based on the lowest estimated p-value of the predictor among all PE_t_/SER_t_ combinations tested.

## Results

### Patient Characteristics

Of the initial 68 patients, four were excluded from the final analysis: two did not undergo surgery, one presented with suboptimal MR images and one had metastatic disease prior to the completion of treatment. In the remaining 64 patients, the median age was 48 (range: 30 to 72; inter-quartile: 14). There were 38 (59%) pre-menopausal (age < 50) patients and 26 (41%) post-menopausal (age ≥ 50). Immunohistochemistry rendered the level of receptor expressions. For breast cancer subtypes, there were 21 (33%) HR+/HER2-, 15 (23%) HER2+, which included HR+/HER2+ and HR-/HER2+, and 11 (17%) TN. The remaining 17 (27%) patients were unknown because their subtype information was not available at the time of the study. In this cohort of 64 patients, 17 patients received taxane after AC treatment (1 HR+HER2-, 6 HER2+, 1 TN and 9 unknown). There were 25 recurrent patients (8 local and 17 distant) with median time-to-recurrence of 22.7 months, and 35 non-recurrent patients with median follow-up time of 87 months. Histologic data and pathologic grade for breast tissue of the same patient cohort has previously been reported [16]. Patients’ demographic and breast cancer subtype characteristics are summarized in Table 1.

**Table 1:** Demographic and patient characteristics.

Patient characteristics	Number of patients (%), Total n = 64
**Age at diagnosis**	
Range 30−72, median 48	
< 50	37 (58)
≥ 50	27 (42)
**Breast cancer subtype**	
HR+/HER2-	21 (33)
HER2+	15 (23)
TN	11 (17)
Unknown	17 (27)
**Recurrence**	n = 25
Local	8 (32)
Distant	17 (68)
Median time to recurrence = 27.7 months (n = 25)	
Median RFS* time = 87 months (n = 39)	

* RFS = recurrence-free survival or follow up time

### Effect of PE and SER Thresholds in the Full Cohort

**Fig 1:**
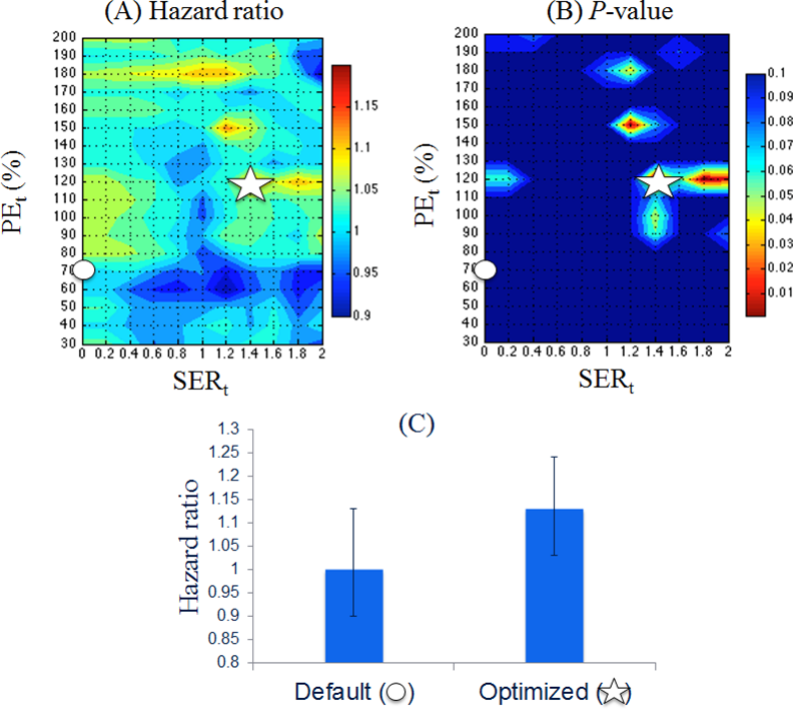
Hazard ratios and p-values estimated for ΔFTV_2_ in the full cohort. (A) The heat map of estimated hazard ratios plotted with PE_t_ in the y-axis from 30% to 200% and SER_t_ in the x-axis from 0 to 2. Hazard ratios within the range of 0.9 to 1.2 were color-coded from blue to red. (B) The heat map of p-values, with estimated hazard ratios shown in (A), is shown in the range of 0.001 to 0.1 with color coded from red to blue. Note that values with high hazard ratios and low p-values are coded in red. On both maps, the default setting with PE_t_ = 70% and SER_t_ = 0 was marked as a circle, and the optimized setting at PE_t_ = 120% and SER_t_ = 1.4 was marked as a star. (C) A plot comparing the estimated hazard ratios and confident intervals at default (hazard ratio (H) of 1.0, 95% CI (0.9–1.1), *p* = 0.88) and optimized (hazard ratio (H) of 1.1, 95% CI (1.0–1.2), *p* = 8 × 10^−4^) settings.

MRI data was analyzed at the baseline, MRI_1_ (n = 64), early treatment, MRI_2_ (n = 50) and pre-surgical after NACT MRI_f_ (n = 64). The smaller sample size at MRI_2_ was due to missing exams and poor image quality. Fig 1 shows the heat maps of estimated hazard ratios and p-values for associations between ΔFTV_2_ and RFS under the influence of PE_t_ and SER_t_ for the full cohort. As shown in Fig 1A and 1B, regions color-coded with high hazard ratios and low p-values were shown in red. Default setting with PE_t_ = 70% and SER_t_ = 0 was marked as a circle, and the optimized setting at PE_t_ = 120% and SER_t_ = 1.4 was marked as a star. The comparison of hazard ratios estimated for ΔFTV_2_ at the default and optimized PE_t_/SER_t_ settings was shown in Fig 1C. Heat maps for the other two predictors, ΔFTV_f_ and FTV_f_, were also generated in the similar fashion. Hazard ratios estimated at the optimized and the default PE_t_/SER_t_ in the full cohort are given in Table 2 for all predictors (ΔFTV_2_ / ΔFTV_f_ / FTV_f_). All hazard ratios were shown along with Wald 95% CIs and p-values.

**Table 2:** Cox proportional hazards ratios at PE_t_/SER_t_ with optimized association with RFS and at the default setting for the full cohort.

	ΔFTV_2_	ΔFTV_f_	FTV_f_
**Optimized**	PE_t_ = 120% / SER_t_ = 1.4	PE_t_ = 100% / SER_t_ = 0.6	PE_t_ = 40% / SER_t_ = 0
	H*: 1.1	H: 1.2	H: 1.1
	CI: 1.0−1.2	CI: 1.1−1.3	CI: 1.05−1.13
	*p* = 8 × 10^−4^	*p* = 7 × 10^−5^	*p* = 7 × 10^−7^
**Default**	PE_t_ = 70% / SER_t_ = 0	PE_t_ = 70% / SER_t_ = 0	PE_t_ = 70% / SER_t_ = 0
	H: 1.0	H: 1.2	H: 1.1
	CI: 0.9−1.1	CI: 1.1−1.4	CI: 1.05−1.13
	*p* = 0.88	*p* = 0.006	*p* = 2 × 10^−6^

H*: hazard ratio

The early treatment FTV percentage change between MRI_1_ and MRI_2_ (ΔFTV_2_) exhibited five PE_t_/SER_t_ combinations resulting in *p* < 0.05, among which the lowest *p* was found at PE_t_ = 120% and SER_t_ = 1.4. Compared to ΔFTV_2_, the final percent change (ΔFTV_f_) showed more PE_t_/SER_t_ combinations that resulted in *p* < 0.05. These threshold combinations were mostly confined to the lower SER_t_ (0–1.0) and lower PE_t_ (30–110%) (Table 2). PE_t_/SER_t_ combinations in the region of PE_t_ from 50 to 100% and SER_t_ from 0.0 to 0.8 resulted in higher estimated hazard ratios (> 1.10) and lower p-values (< 0.001). This optimal region of thresholds was consistent with previous findings [16]. At MRI_f_, FTV_f_ showed the most robust association with RFS among the three predictors with *p* < 0.05 across all measured combinations of PE_t_/SER_t_ (Table 2).

### Effect of PE and SER Thresholds in Breast Cancer Subtypes

**Fig 2:**
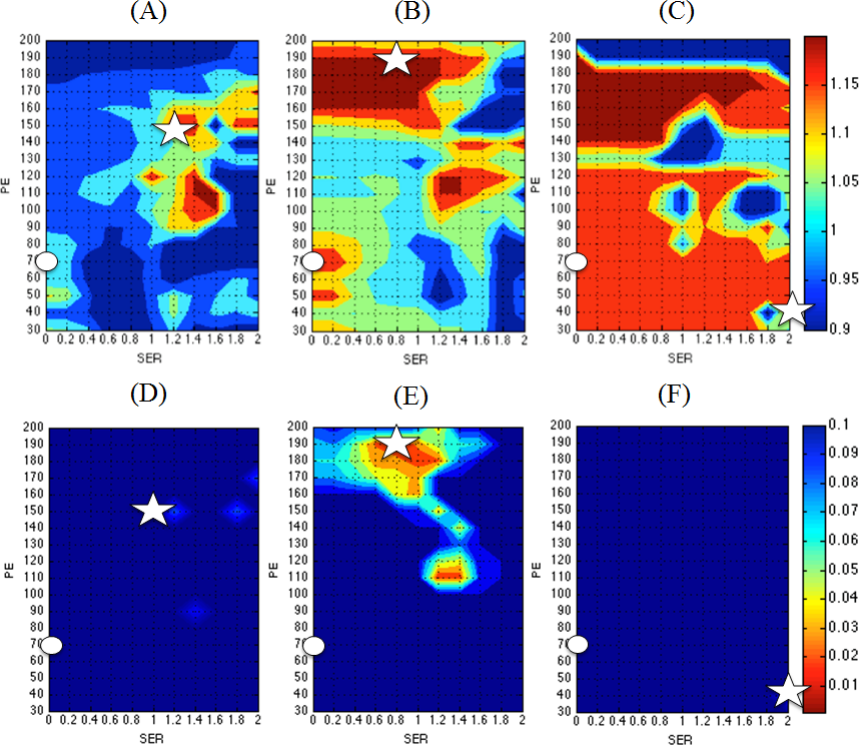
Heat maps for estimated hazard ratios (top panels) and p-values (bottom panels) of ΔFTV_2_ in breast cancer subtypes. HR+/HER2-: hazard ratios (A) and p-values (D); HER2+: hazard ratios (B) and p-values (E); TN: hazard ratios (C) and p-values (F). Note that values with high hazard ratios and low p-values are coded in red. On all maps, the default settings with PE_t_ = 70% and SER_t_ = 0 were marked as circles, and the optimized PE_t_ and SER_t_ settings were marked as stars.

Estimations of FTV and RFS associations under the influence of PE_t_ and SER_t_ in breast cancer subtypes were plotted with heat maps similar to that of the full cohort. For consistency, we only show heat maps for ΔFTV_2_ in each subtype (Fig 2). The corresponding optimized PE_t_/SER_t_ combinations were also marked as stars and default as circles.

**Fig 3:**
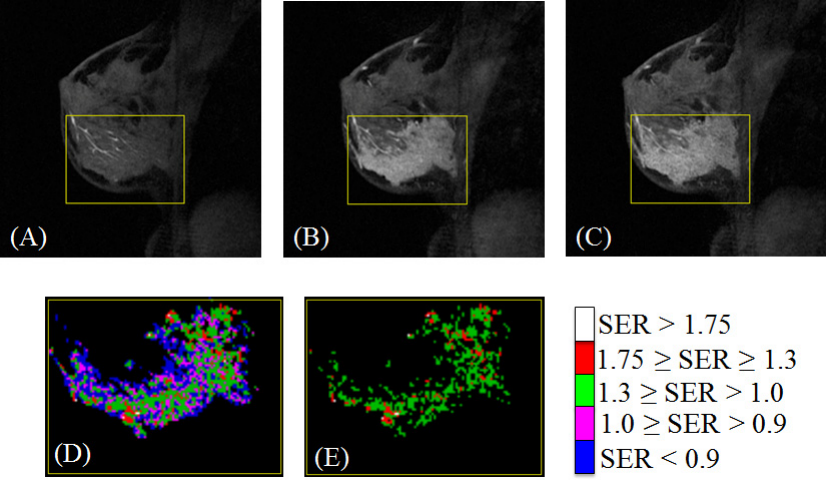
Representative clinical case showing the effect of changing PE_t_ and SER_t_ in FTV calculation. (A)−(C): representative baseline MR images of breast cancer. Yellow rectangular indicates tumor ROI. (A) was acquired before contrast injection. (B) and (C) were at the same slice location as in (A) but was acquired at the early and late enhancement time points after contrast injection. (D) and (E) show voxels selected within the ROI using different PE_t_ / SER_t_ combinations. SER colormap is shown on the right of (E). (D) PE_t_ = 70%, SER_t_ = 0, FTV = 43.4 cc. (E) PE_t_ = 100%, SER_t_ = 1.0, FTV = 20.6 cc.

For patients with HR+/HER2- breast cancer (n = 21), ΔFTV_f_ had only one PE_t_/SER_t_ combination (PE_t_ = 100% and SER_t_ = 1.0) that resulted in *p* < 0.05. In this subtype, neither FTV_f_ nor ΔFTV_2_ had statistically significant (*p* < 0.05) associations with RFS using all PE_t_/SER_t_ combinations tested (Fig 2A and 2D). Estimated hazard ratios with confident interval and p-values at the optimized PE_t_/SER_t_ combination and at default setting for this subtype are listed in Table 3. A clinical example of applying the default PE_t_/SER_t_ and the thresholds obtained by optimization in this subtype was shown in Fig 3.

**Table 3:** Cox proportional hazards ratios at PE_t_/SER_t_ with optimized association with RFS and at default setting for patients with HR+/HER2- breast cancer.

	ΔFTV_2_	ΔFTV_f_	FTV_f_
**Optimized**	PE_t_ = 150% / SER_t_ = 1.2	PE_t_ = 100% / SER_t_ = 1.0	PE_t_ = 110% / SER_t_ = 1.6
	H: 1.3	H: 1.2	H: 27
	CI: 1.0−1.8	CI: 1.0−1.3	CI: 0.2−3624
	*p* = 0.08	*p* = 0.04	*p* = 0.2
**Default**	PE_t_ = 70% / SER_t_ = 0	PE_t_ = 70% / SER_t_ = 0	PE_t_ = 70% / SER_t_ = 0
	H: 1.0	H: 0.9	H: 1.0
	CI: 0.7−1.5	CI: 0.6−1.3	CI: 0.8−1.3
	*p* = 0.9	*p* = 0.6	*p* = 0.9

In the subset of HER2+ (n = 15) patients, FTV_f_ had statistically significant (*p* < 0.05) associations with RFS in the full range of PE_t_ (30–200%) and lower range of SER_t_ (0–1.0). Meanwhile, very few PE_t_/SER_t_ combinations resulted in associations with RFS for which *p* < 0.05 was observed for both ΔFTV_2_ and ΔFTV_f_ (See Fig 2B and 2E for the results of ΔFTV_2_ presented in heat maps and Table 4 for optimized and default estimations).

**Table 4:** Cox proportional hazards ratios at PE_t_/SER_t_ with optimized association with RFS and at the default setting for patients with HER2+ breast cancer.

	ΔFTV_2_	ΔFTV_f_	FTV_f_
**Optimized**	PE_t_ = 190% / SER_t_ = 0.8	PE_t_ = 190% / SER_t_ = 0.4	PE_t_ = 140% / SER_t_ = 0.2
	H: 1.7	H: 26	H: 3.8
	CI: 1.1−2.6	CI: 2−423	CI: 1.1−12.6
	*p* = 0.02	*p* = 0.02	*p* = 0.03
**Default**	PE_t_ = 70% / SER_t_ = 0	PE_t_ = 70% / SER_t_ = 0	PE_t_ = 70% / SER_t_ = 0
	H: 1.2	H: 1.2	H: 1.3
	CI: 0.9−1.6	CI: 0.8−1.8	CI: 1.0−1.5
	*p* = 0.3	*p* = 0.4	*p* = 0.03

In the 11 patients with TN breast cancer, Cox proportional hazard analysis showed *p* < 0.05 associations between FTV_f_ and RFS in the lower range of PE_t_ (30–120%) and lower range of SER_t_ (0–0.8). The lowest p-value was found at the pair PE_t_ = 130% and SER_t_ = 0.8. A larger range of PE_t_ (30–140%) and SER_t_ (0–1.2) resulted in associations of ΔFTV_f_ and RFS with *p* < 0.05. No combination of PE_t_/SER_t_ resulted in *p* < 0.05 for ΔFTV_2_ in this subset (Fig 2C and 2F). Resulting optimized and default estimations for this subtype can be found in Table 5.

**Table 5:** Cox proportional hazards ratios at PE_t_/SER_t_ with optimized association with RFS and at the default setting for patients with TN breast cancer.

	ΔFTV_2_	ΔFTV_f_	FTV_f_
**Optimized**	PE_t_ = 40% / SER_t_ = 2.0	PE_t_ = 110% / SER_t_ = 1.2	PE_t_ = 130% / SER_t_ = 0.8
	H: 1.4	H: 1.2	H: 2.9
	CI: 0.95−2.1	CI: 1.0−1.4	CI: 1.2−6.8
	*p* = 0.09	*p* = 0.01	*p* = 0.02
**Default**	PE_t_ = 70% / SER_t_ = 0	PE_t_ = 70% / SER_t_ = 0	PE_t_ = 70% / SER_t_ = 0
	H: 1.5	H: 1.3	H: 1.1
	CI: 0.9−2.6	CI: 1.0−1.7	CI: 1.0−1.1
	*p* = 0.1	*p* = 0.02	*p* = 0.02

## Discussion

Imaging plays a critical role in monitoring tumor regression during NACT. Empirical DCE-MRI parameters representing both contrast agent enhancement and washout were shown to have significant correlation with recurrence free survival (RFS) for patients going through NACT [14]. Furthermore, tumor volume measured in DCE-MRI by setting empirical parameters [13] has not only allowed for quantitative assessment of tumor response to treatment, change in tumor volume pre- vs. post-treatment has also been shown to be a strong predictor of RFS [12,13].

A prior study demonstrated that the ability of functional tumor volume change (ΔFTV) to predict RFS was improved by optimizing MR contrast-enhancement thresholds [16]. The prior study evaluated ΔFTV_f_ as a single predictor. It is now known that although pathologic complete response (pCR) is a good predictor of RFS in the neoadjuvant setting of higher risk breast cancers, pCR is a better predictor of RFS by subtype than for all subtypes combined [22]. In addition, final MRI volume is a better predictor of residual tumor for TN than HER2+ and HR+/HER2- tumors [15] and different breast cancer subtypes show heterogeneous patterns of response. For all of these reasons, this study investigated additional predictors of outcome measuring FTV change earlier in treatment (ΔFTV_2_) and also final residual FTV (FTV_f_) at the end of the treatment. The additional FTV predictors being explored in this study may also allow us to potentially select a more suitable recurrence risk predictor for each breast cancer subtype.

Indeed from this analysis, we observed a very different recurrence risk profile predicted by ΔFTV_2_ from a wide range of PE_t_/SER_t_ combinations for the full cohort compared to other predictors. In the full cohort, the optimal PE_t_/SER_t_ settings for ΔFTV_2_, chosen by the combination of low p-value and high hazard ratio, were located in the higher PE and SER range, suggesting that the default PE_t_ = 70% and SER_t_ = 0.0 setting may not produce the best early FTV predictor for RFS. Compared to ΔFTV_2_, ΔFTV_f_ showed its predictive association with RFS (*p* < 0.001) in the lower range of PE_t_ (50−100%) and SER_t_ (0−0.8), which also included the default setting above. This finding is in agreement with the PE/SER threshold settings obtained previously [16]. The predictor FTV_f_ showed *p* < 0.05 associations with RFS in all PE_t_/SER_t_ combinations. In the full cohort, these findings indicate that the optimal PE_t_/SER_t_ may be influenced by treatment time point and that the default setting may not produce the most optimal ΔFTV at early time point to access recurrence risk.

In the neoadjuvant setting, although tumor regression measured by MRI using default parameter thresholds is in concordance with pathologic response [12], such concordance varies widely by breast cancer subtype [15] as does its predictability of survival outcome [23]. Based on our efforts on imaging threshold optimization for RFS prediction, we reasoned that such optimization might be applicable to the breast cancer subtype analysis.

When the cohort of 64 patients was analyzed based on receptor expressions, a different hazard ratio / p-values profile emerged. Each breast cancer subtype exhibited a unique distribution of hazard ratios predicted by FTV predictors that were generated from a set of optimal PE_t_/SER_t_ combination. For example, only HER2+ showed a few PE_t_/SER_t_ combinations that were significantly associated with RFS for ΔFTV_2_ as a predictor. And for ΔFTV_f_, both HER2+ and TN showed combinations that had statistically significant associations with RFS, while HER2+ appeared in the higher range of PE. HR+HER2- did not show any significant associations with RFS for the entire ranges of PE and SER, althoughit could be due to the small sample size. HER2+ was in the lower range of SER (< 0.8) with the full range of PE being tested.

Given the heterogeneity of breast cancer, each subtype molecular signature such as growth factors [24] and matrix metalloproteinases [25] may alter its tumor vascularity [26] that may be reflected by the subtle difference in MR contrast enhancement. Our findings here further substantiate the distinctive characteristic of breast cancer subtypes, not only by pathological analysis but also by imaging.

The current retrospective study has a few limitations. First, the dataset was acquired between 1995−2002 with breast cancer patients receiving adriamycin-cytoxan and the taxane-based regimen as the standard NACT. Clinical guidance of NACT may have changed, but our study is not limited to specific treatment as long as all patients are treated the same. In this cohort, of the 17 patients receiving taxane-based treatment, 1 was HR+HER2-, 1 was TN, and 6 were HER2+ (60% of the subgroup). Therefore, the prediction of RFS in the HER2+ group may have been biased by a relatively large portion of patients were treated with taxane. As shown previously, RFS is affected by tumor characteristics such as tumor size, pathological size and lymph node status [27]. Due to the small sample size, we were not able to evaluate these characteristics in each subtype. Further study using a larger cohort is underway. In this study, the chemotherapy that patients received largely preceded the use of HER2 targeted agent. This may influence our evaluation of RFS in the HER2+ cohort by today’s standard of treatment. Another limitation is that the sample size was small and was limited when the cohort was further divided into subtypes, which limited the subtyping options. For example, the number of patients in HER2+ subtype would reduce to less than 10 if we further divide HER2+ patients into HR+HER2+ and HR-HER2+ subgroups. Therefore, the resulting prediction and optimal PE_t_/SER_t_ settings by subtype should be interpreted with caution. We acknowledged that patients with multi-focal diseases would be prone to sampling error for subtype classification. We recognized that the prediction and PE/SER threshold optimal setting for the entire cohort might be influenced by the larger sample size of the HR+/HER2- group. Other than hormone receptor status and HER2 status, breast cancer can be further subset into luminal, basal-like, normal-like, and erbB2+ based on the molecular classification proposed by Perou *et al*. [2]. These subtypes were shown to respond differently to preoperative chemotherapy [28]. Though we only tested a simple classification in this paper, the framework described here can be applied to other classification schemes.

Despite these limitations, this study was undertaken to explore the significance of FTV for predicting breast cancer recurrence following NACT. While this study is retrospective in nature and targeted treatment for breast cancer subtypes has become the standard of care, our findings nevertheless suggest that performance of imaging predictors based on FTV may be improved if threshold optimization is performed separately for the clinically-relevant subtypes defined by HR and HER2 receptor expression.
